# Common risk factor approach to address socioeconomic inequality in the oral health of preschool children – a prospective cohort study

**DOI:** 10.1186/1471-2458-14-429

**Published:** 2014-05-06

**Authors:** Loc G Do, Jane A Scott, W Murray Thomson, John W Stamm, Andrew J Rugg-Gunn, Steven M Levy, Ching Wong, Gemma Devenish, Diep H Ha, A John Spencer

**Affiliations:** 1 Australian Research Centre for Population Oral Health, The University of Adelaide, Adelaide, Australia; 2 Curtin University, Perth, Australia; 3 University of Otago, Dunedin, New Zealand; 4 University of North Carolina at Chapel Hill, Chapel Hill, NC, USA; 5 University of Newcastle upon Tyne, Newcastle, UK; 6 The University of Iowa, Iowa city, IA, USA

**Keywords:** Children, Early childhood caries, Socioeconomic inequality, Prospective cohort study

## Abstract

**Background:**

Dental caries remains the most prevalent chronic condition in children and a major contributor to poor general health. There is ample evidence of a skewed distribution of oral health, with a small proportion of children in the population bearing the majority of the burden of the disease. This minority group is comprised disproportionately of socioeconomically disadvantaged children. An in-depth longitudinal study is needed to better understand the determinants of child oral health, in order to support effective evidence-based policies and interventions in improving child oral health. The aim of the Study of Mothers’ and Infants’ Life Events Affecting Oral Health (SMILE) project is to identify and evaluate the relative importance and timing of critical factors that shape the oral health of young children and then to seek to evaluate those factors in their inter-relationship with socioeconomic influences.

**Methods/Design:**

This investigation will apply an observational prospective study design to a cohort of socioeconomically-diverse South Australian newborns and their mothers, intensively following these dyads as the children grow to toddler age. Mothers of newborn children will be invited to participate in the study in the early post-partum period. At enrolment, data will be collected on parental socioeconomic status, mothers’ general and dental health conditions, details of the pregnancy, infant feeding practice and parental health behaviours and practices. Data on diet and feeding practices, oral health behaviours and practices, and dental visiting patterns will be collected at 3, 6, 12 and 24 months of age. When children turn 24-30 months, the children and their mothers/primary care givers will be invited to an oral examination to record oral health status. Anthropometric assessment will also be conducted.

**Discussion:**

This prospective cohort study will examine a wide range of determinants influencing child oral health and related general conditions such as overweight. It will lead to the evaluation of the inter-relationship among main influences and their relative effect on child oral health. The study findings will provide high level evidence of pathways through which socio-environmental factors impact child oral health. It will also provide an opportunity to examine the relationship between oral health and childhood overweight.

## Background

While dental caries is largely preventable [1] and there are effective population-based and individual preventive strategies such as community water fluoridation, brushing with fluoridated toothpaste, and widespread access to dental care, dental caries remains the most prevalent chronic disease in children, exerting significant impact on both the children affected and society in general. The prevalence of dental caries in primary school children in Australia [2] and in the U.S. [3,4] is four to five times higher than that of asthma, the next most prevalent chronic condition in this age group. Furthermore, dental caries is the most common (yet avoidable) cause of acute hospital admissions in children [5] due to the common situation of children requiring multiple dental extractions under general anaesthesia. Dental caries has a major financial impact on the health system and psychological impact on the children and their families [6].

A summary of the latest key findings on the state of oral health of the Australian population estimates the prevalence of early childhood caries (ECC) among five year-old Australians to be 42% [7]. Despite a substantial level of resources being directed to prevention and dental services for children in Australia, the oral health of Australian children has worsened [2], with inequality in oral health widening in the last decade. The extent of caries experience among 6-year-old Australian children increased by 24% during the late 1990s [2]. More alarmingly, there is ample evidence of a skewed distribution of dental caries with a small proportion of children in the population bearing the majority of the burden of the disease [8]. The most severely affected 10% of 4-year-old Australian children had (on average) seven teeth with dental caries experience [2], a level that significantly impacts on their lives [9]. This minority group is comprised mostly of socioeconomically disadvantaged children.

The pathways, through which socioeconomic disadvantage affects child oral health, remain unclear. Children of different socioeconomic backgrounds could have different patterns of food and fluid consumption and different patterns of oral health practices such as toothbrushing from a very early age. Such differences could act as mediators of the association between socioeconomic disadvantage and child oral health. Understanding the mediation process is necessary to better address the oral health inequality and improve overall child health. It is also important to note that the evidence base on measures to tackle oral health inequalities is limited. These important issues require in-depth research to understand the determinants of child oral health and their inter-relationship to support effective evidence-based policies and interventions to improve child oral health, a major contributor to general health.

### Aim and objectives

The research project described in this paper has been funded by an Australian National Health and Medical Research Council (NHMRC) Project Grant # 1046219 (2013-2016) under the scientific title ‘*Common risk factor approach to address socioeconomic inequality in oral health of contemporary Australian preschool children*’. The aims of the project, henceforth referred to by its working title “*Study of Mother’s and Infant’s Life Events Affecting Oral Health (SMILE)*” project, are to identify and evaluate the relative importance and timing of critical factors that shape the oral health of young children and then to seek to evaluate those factors in their inter-relationship with socioeconomic influences. The knowledge gained in this study will point to key factors that differentiate the oral health of children from different socioeconomic groups, leading to strategies for the improvement of oral health in disadvantaged young children.

This study has four specific objectives:

1. To measure socioeconomic variations in dietary patterns including patterns of fluid consumption, patterns of oral health practices such as toothbrushing and fluoride use and non-fluoride preventive applications of children from birth to age two years;

2. To evaluate variations in child oral health status at age two years;

3. To evaluate the timing and extent to which health-promoting factors attenuate the effect of disease-predisposing factors and;

4. To evaluate the commonality of risk factors for oral health and obesity, a highly prevalent childhood general health condition related to infant feeding practice.

## Methods

### Study design

The SMILE project will apply an observational prospective study design to follow a cohort of socioeconomically-diverse South Australian newborns and their primary care-givers (mothers will be used in this paper from here on), from birth until they reach toddler age. A multivariable, multilevel approach will be applied to data collection and analysis. The timeline and main groups of outcome and explanatory variables are outlined in Table 1.

**Table 1:** Overview of variables in the study

**Time**	**Variables**	**Collection mode**
**Birth**	Community-level SES	ABS databases
	Parental SES	Self-complete questionnaires and face-to-face interview
	Parental general and dental health	
	Infant feeding practices	
	Child birth weight	
**Age 3 months**	Infant Feeding Practices	Self-complete questionnaire or telephone interview
	General health conditions	
	Primary Care Givers’ Dental Health Beliefs and Attitudes	
**Age 6 months**	Infant Feeding Practices for 3–6 months period	Same as for 3 months
	General health conditions	
	Information on oral health care advice	
**Age 12 months**	Estimated intake of free sugars and fluoride	Self-complete short questionnaire or telephone interview and 24 hour recall interview and 2 day diet record
	Child teeth present	
	Oral health practices	
	Dental visit	
**Age 24–30 months**	Community-level SES	ABS databases
	Parental information	Same as for birth
	PCG oral health status	Clinical examination
	Oral hygiene status	
	Dental caries experience	
	Periodontal status	
	Estimated intake of free sugars and fluoride	Self-complete short questionnaire and food frequency questionnaire
	Child teeth present	
	Oral health practices	
	Dental visit	
	Child oral health status:	Clinical examination
	Oral hygiene status	
	Dental caries experience	
	Developmental defects	
	Child and PCG microbiological assessment	Salivary plaque samples
	Child anthropometric assessment	Measurements

ABS: Australian Bureau of Statistics.

The project has received ethical approval from the Southern Adelaide Clinical Human Research Ethics Committee (HREC # 50.13, approval date: 28 Feb 2013), the South Australian Women and Children Health Network (HREC # HREC/13/WCHN/69, approval date: 7 Aug 2013) and clinical governance clearance from the three participating maternity hospitals in Adelaide, Australia.

The study participant recruitment has commenced since July 2013 and is expected to last 12 months. The 3-month and 6-month data collection is ongoing.

#### ***Targeted population***

The study setting is Adelaide and its immediate surroundings. The targeted population is all children born in Adelaide in 2013-14. Annual statistics compiled by the Pregnancy Outcome Unit, Department of Health of South Australia show that 20,344 children were born in 2011 in Adelaide, with around 60% of all live births occurring in the three largest metropolitan public hospitals providing maternity services to the whole of Adelaide. The cohort will be recruited from these three public hospitals.

Recruitment began in mid-2013 and will continue to mid-2014. All new mothers at the postnatal departments who are able to understand the description and instructions of the study are invited to participate. The mothers who indicate their intention to move out of South Australia within a year are excluded. Premature or low birth weight children can be included if mothers agree to participate.

#### ***Sample size calculation***

The required baseline sample size was calculated to address the most sensitive objective (Aim 2) of this four-year study using standard methods [10]. Sample size was calculated to detect a rate ratio of 0.2 between slopes (considered a small effect size [10]) for explanatory variables in multivariable regression models for caries experience (mean dmfs) at age two years (Aim 2) with an alpha level of 0.05 (two-tail) and statistical power of 90%. The calculated sample size required at age two years is 1,398 children. A minimum two-year retention rate of 80% was used in the calculation. It resulted in a targeted sample size at birth of 1,677 children (rounded up to 1,700). This recruitment target is highly achievable given the population pool of 16,000 children. The expected two-year retention rate of 80% is considered conservative based on our experience [11]. Using this sample size, statistical power expected to achieve other aims was calculated at over 90% after taking into account possible interaction among variables.

#### ***Sample recruitment***

Research workers experienced in working with women and small children such as dental therapists or dental nurses have been employed and trained to recruit participants for the study. An information package about the study has been developed for the potential participants. Small participation incentives (e.g. toothbrush and tooth paste samples) and travel reimbursements will be provided to participants to improve recruitment and retention rates.

The research workers visit the hospitals on a frequency determined by the birth rate at each hospital. Contact is made with mothers in the maternity ward, typically within 48 hours of the birth of their infant. Mothers are provided with a written and verbal explanation about the study and then invited to participate in the study. All questions are answered and informed consent obtained. Low-SES mothers will be oversampled, as recommended for prospective cohort studies [12]. This can be done by increasing the sample from those hospitals who primarily service this group. Distribution of participants by area-level SES and individual SES distribution will be checked frequently and compared with the population parameters. Oversampling low-SES groups will offset for expected relatively higher attrition rate by these groups. As the study progresses, sample maintenance procedures such as birthday cards to the child will be used. Response rates by different SES groups at data collection rounds will also be checked frequently. If necessary, different strategies will be applied to SES sub-groups to improve overall response rate.

#### ***Primary outcome variable of oral health status***

After children reach age two years (age range 24–30 mo), they and their mothers will be invited to undergo a dental examination conducted by a small group of specially trained dentists under standardised clinical conditions. Standard clinical indices, developed at the Australian Research Centre for Population Oral Health (ARCPOH) based on the US National Institute of Dental and Craniofacial Research (NIDCR) protocol [13] and the International Caries Detection and Assessment System (ICDAS-II) [14], will be used. The ARCPOH protocol is currently being used in a National Child Oral Health Survey. Two principal investigators of this cohort study are principal investigators and main examiner trainers for that survey. Non-cavitated or cavitated carious lesions, filling, missing tooth surfaces because of decay, non-carious developmental defects such as hypoplasia and gingival conditions will be recorded. Five per cent of children and mothers will be randomly selected for a replicate examination to determine inter- and intra-examiner reliability. Salivary plaque samples will be collected from both mothers and children. Samples will be stored at the University of Adelaide laboratory for analysis of cariogenic bacteria.

#### ***Outcome variable of child weight status***

At the examination children will be weighed and measured using standardised equipment and methodology [15]. Child weight status will be assessed based on World Health Organization age and gender specific BMI percentiles, where a BMI >85^th^ and <97^th^ percentile is overweight and ≥97^th^ percentile is obese (http://www.who.int/childgrowth/standards/en/.)

#### ***Main explanatory variables***

Mother- and child-related information will be collected using a series of self-completed postal or online questionnaires and face-to-face interviews during the study period. The first wave of questionnaire data collection is at recruitment, with subsequent waves occurring at 3, 6, 12 and 24 months.

#### ***Community-level factors***

Area-based Census data reported by the Australian Bureau of Statistics will be obtained and linked with mothers’ residence. The Socioeconomic Index for Areas (SEIFA) (Australian Bureau of Statistics 2011) will be used to classify participants into groups by area-level SES. Any subsequent changes in residential locations will be collected.

#### ***Mother and family-related information***

Mothers’ socioeconomic details such as age, income, education and occupation will be collected using standardised questionnaires developed at ARCPOH [16]. Household socioeconomic indicators at recruitment with updates at subsequent data collections form the SES characteristics. Data on general and oral health knowledge and oral health beliefs and practices during the prenatal period and during the study period will be periodically collected. Mothers will be offered an oral health examination at the time of examination for children at age two years. During the examinations at age two years, face-to-face interviews with the mothers will be conducted to collect more complex information. Maternal oral health beliefs, status, microbiological assessments and self-reported oral health practices will be used as explanatory variables in the analyses.

#### ***Child-related information***

Parents will be asked to report birth and subsequent general health issues. Data on dietary patterns, patterns of fluid consumption, and oral health practices are to be collected at 3, 6, 12 and 24 months. The collected information will be used to estimate fluoride exposure and intake. Details of receipt of oral health anticipatory guidance or oral health advice from dental professionals and general health practitioners including Child and Youth Health nurses, will be obtained from the questionnaires and interviews. Data on the timing, reason, nature and consequences of dental visits will be sought.

#### ***Dietary patterns and patterns of fluid consumption***

Dietary data will be collected at age 3, 6, 12 and 24 months. Information on infant feeding patterns (including an estimation of breast milk and/or infant formula intake) will be collected using questionnaires previously used in the first and second Perth Infant Feeding Studies [17,18] and the Australian National Infant Feeding Study [19]. A particular focus will be the collection of information related to the practice of prolonged breastfeeding and night time breastfeeding.

Detailed information on usual food and beverage intake and dietary behaviours will be collected at 12 months using a telephone 24 hour recall interview conducted by a trained nutritionist supplemented with a 2-day (one week and one weekend day) food record completed by the mother [20]. The food record booklet, which will include detailed instructions and a food measurement aid (including photos of infant feeding bowls, cups and utensils), will be mailed along with the 12 month questionnaire. A cover letter will advise mothers that an interviewer will ring them within 7-10 days to collect information on what their child is eating and explain how to complete the food record. On return of the food record an interviewer will contact the mothers by telephone to clarify any record with incomplete information (such as missing estimates of intake, or time of consumption). At 24 months dietary information will be collected using a short food frequency questionnaire, specifically designed and validated for the purposes of this study. Dietary data will be analysed using the FoodWorks^®^ (Xyris Software) computerised dietary assessment program.

#### ***Estimation of intake of free sugars and fluoride intake***

If required, fluoride concentrations in the main foods and beverages reported in the diet record will be assayed in our Adelaide laboratory using methods employed successfully in our previous studies [21,22]. Estimates of fluoride intake will be based on the fluoride concentrations in foods and beverages, and the amount consumed [23,24]. The type, amount and frequency of intake of free sugars will be determined from the 24 hour recall and 2 day estimated diet record at 12 months and the food frequency at 24 months questionnaire using the method described by Kelly and colleagues [25].

Consumption of public water will be collected in detail using questionnaires and interviews at age 3, 6, 12 and 24 months. This information will be linked with a database of fluoride levels in public water source for residential locations. The proportion of tap water in total fluid intake and use of any fluoride-removing filter will be detailed. The level of fluoride in public mains water will be measured periodically in the laboratory. If participants report using other sources of water, such as bottled water or rain water or using water filter at home, water samples will be obtained and fluoride levels measured.

#### ***Oral health practices***

The questionnaires will collect information on the oral health practices of the children at age 6, 12 and 24 months. This will include age of commencement of tooth cleaning without toothpaste, when fluoridated toothpaste use commences, and components of toothbrushing practice such as frequency of brushing, toothpaste type, toothpaste amount used per brushing, eating/licking toothpaste habit and rinsing/spitting after brushing. Information on professionally-applied fluoride applications (such as fluoride varnish) will be collected, noting the frequency, type, and age when it is used. Use of fluoride supplements, age of use and dosage will also be queried. Occurrence of dental visits and dental care received by the children will be collected.

Information on visiting for dental reasons will be collected. Places of visit, age when the visits begin, reasons for the visit, oral health advice, preventive procedures and treatment received will be documented.

#### ***Fluoride exposure and intake from oral health practices***

Fluoride exposure and intake from oral health practices will be estimated using the collected data. Type of toothpaste, amount of toothpaste per brushing, frequency of brushing, proportion of ingested toothpaste, use of fluoride supplements and other in-office fluoride applications will be used to estimate fluoride intake [23,24]. Body weights will be used to calculate average fluoride intake per kg body weight from both dietary intake and oral health practices.

#### ***General health conditions of mothers and children***

Information on a number of common health conditions of the children will be collected using questionnaires. Use of antibiotics and sugar-containing medications will be detailed. Information on mothers’ general health condition will be collected through questionnaires. Mothers will be asked to bring the child’s Child and Youth Health record book (Blue book) to the oral examination. Permission will be asked to transcribe information of the child’s development and general health during the period.

#### ***Analysis and reporting***

We will evaluate Objectives 1 to 4 using SAS (SAS Institute Inc., Cary, NC, USA) and the multilevel analytical software, MLwiN (The Centre for Multilevel Modelling, the University of Bristol, UK). Potentially, count of dmfs scores can be analysed using a negative-binomial link function with adjustment (using generalized estimating equations in SAS PROC GENMOD) to account for within-person clustering of observations [26]. If the caries outcome data result in a large excess of zero counts (particularly at the 2-year period), we will adopt models based on zero-inflated negative binomial (ZINB) regression. Random effect models will be generated using SAS and MLwiN [27,28]. A number of covariates are time-varying. Therefore, it will be appropriate to apply marginal structural models (MSM) to obtain estimates of causal inferences between those factors and the outcomes [29,30].

We will report the results of the objectives as peer-reviewed scientific publications following the STROBE guidelines [31] for reporting observational research. Results will be disseminated to appropriate health agencies in Australia and internationally through publications, conferences and workshops.

## Discussion

This prospective cohort study will examine a wide range of determinants influencing child oral health and a number of related general conditions. It will lead to the evaluation of the inter-relationships among groups of main influences and their relative effect on oral health in a representative sample of children. The study findings will provide high level evidence of pathways through which socio-environmental factors impact the oral health of young children. The study will also provide opportunity to examine the relationship between oral health and childhood obesity and their shared pathways. We believe that the evidence will enable a more effective common risk factor approach [32] to tackle these two common childhood problems. The common risk factor approach is capable of reducing social inequalities by focusing on improving health conditions in general for the whole population and for groups at high risk and of integrating oral health into general health through a Social Determinants framework [33]. From there, appropriate interventions will be identified to tackle socioeconomic inequality in child oral health and, hence, to improve the overall oral health and general health of the child population.

## Competing interests

The authors declare that they have no competing interests.

## Authors’ contributions

LD and AJS conceived the overall design of the study. JAS, WMT, JWS, ARG and SML contributed to development of the study design and data collection instruments. GD, CW and DHH contributed to the development of data collection instruments. All authors contributed to writing and approval of this paper.

## Pre-publication history

The pre-publication history for this paper can be accessed here:

http://www.biomedcentral.com/1471-2458/14/429/prepub
